# Liver microbial community and associated host transcriptome in calves with feed induced acidosis

**DOI:** 10.3389/fvets.2023.1193473

**Published:** 2023-10-23

**Authors:** Wenli Li, Anna Larsen, Brianna Murphy, Priscila Fregulia

**Affiliations:** ^1^US Dairy Forage Research Center, USDA-Agricultural Research Service, Madison, WI, United States; ^2^Department of Animal and Dairy Sciences, University of Wisconsin-Madison, Madison, WI, United States; ^3^Oak Ridge Institute for Science and Education, Oak Ridge, TN, United States

**Keywords:** feed-induced acidosis, liver microbiome, liver transcriptome, RNA sequencing, young calves

## Abstract

**Introduction:**

In the dairy industry, calves are typically fed diets rich in highly fermentable carbohydrates and low in fibrous feeds to maximize ruminal papillae and tissue development. Calves on such diets are vulnerable at developing ruminal acidosis. Prevalent in cattle, liver abscess (LA) is considered a sequela to ruminal acidosis. LAs can cause significant liver function condemnation and decreased growth and production. Currently, we know little about the liver microbiome in calves with feed-induced acidosis.

**Methods:**

Using our established model of ruminal acidosis, where young calves were fed an acidosis-inducing (AC) or -blunting (control) diet starting at birth until 17-week of age, we investigated microbial community changes in the liver resultant from ruminal acidosis. Eight calves were randomly assigned to each diet, with four animals per treatment. Rumen epithelium and liver tissues were collected at 17 weeks of age right after euthanasia. Total RNAs were extracted and followed by whole transcriptome sequencing. Microbial RNA reads were enriched bioinformatically and used for microbial taxonomy classification using Kraken2.

**Results:**

AC Calves showed significantly less weight gain over the course of the experiment, in addition to significantly lower ruminal pH, and rumen degradation comparison to the control group (*p* < 0.05). In the liver, a total of 29 genera showed a significant (*p* < 0.05) abundance change (> 2-fold) between the treatments at 17-week of age. Among these, Fibrobacter, Treponema, Lactobacillus, and Olsenella have been reported in abscessed liver in cattle. Concurrent abundance changes in 9 of the genera were observed in both the liver and rumen tissues collected at 17-week of age, indicating potential crosstalk between the liver and rumen epithelial microbial communities. Significant association was identified between host liver gene and its embedded microbial taxa. Aside from identifying previously reported microbial taxa in cattle abscessed liver, new repertoire of actively transcribed microbial taxa was identified in this study.

**Discussion:**

By employing metatranscriptome sequencing, our study painted a picture of liver microbiome in young calves with or without feed induced acidosis. Our study suggested that liver microbiome may have a critical impact on host liver physiology. Novel findings of this study emphasize the need for further in-depth analysis to uncover the functional roles of liver resident microbiome in liver metabolic acidosis resultant from feed-related ruminal acidosis.

## 1. Introduction

One of the most dramatic gastrointestinal tract (GIT) transformations in young calves occurs during the weaning period. This period is frequently associated with impaired GIT health and extreme GIT changes due to drastic diet shifts (1, 2). Dairy calves are born with a rumen environment that is devoid of gut microbes and must be inoculated from their environment, which begins ~within 20 min of birth (3). The developing rumen microbial community and anaerobic environment begins in the first days after birth (4) and is further influenced by multiple early environmental factors including delivery mode (5), early life inoculation (6–8) and early life dietary intervention (9–11).

In the dairy industry, calves are typically managed to maximize ruminal papillae and tissue development by feeding diets that are rich in highly fermentable carbohydrates and low in fibrous feeds (12), due in part, to the enhanced rate and extent of ruminal development that can be achieved from these concentrated diets (13). An unintended consequence of these diets is the damage to the rumen wall due to the excessive production of short chain fatty acids (14). Additionally, increasing rumen acidotic conditions can have detrimental effects on whole animal physiology and growth rate (14, 15). Rumen mucosal damage is one prominent GIT ailment young calves suffer during the calf weaning period (16), which is coupled with sudden gut permeability (17). Increased GIT permeability caused by disruption in tight junction proteins (TJs), including occludin, claudins, junctional adhesion molecules (JAMs), and zonula occludins (ZO) (18) can lead to bacterial translocation from the GIT into the bloodstream (19). The basis for disrupted GIT barrier function involves a number of cytokines, including TNF-α and IFN-γ (20). On the other hand, gut microbes have been shown to direct TJ protein expression and localization in both *in vitro* and *in vivo* models (21) via the release of peptides, toxins (22, 23), or metabolites (24–26).

Prevalent in cattle, liver abscesses (LAs) are considered ancillary to ruminal acidosis. LAs can cause significant economic loss as the result of liver function condemnation and decreased growth and production (27, 28). The detrimental impact of LA on animal growth was first reported by 29, who discussed the incidence of LA and its contribution to reduced viscera yields. Of cattle slaughtered in 1940, the liver abscess rate was 5.5%. Since then, the reported incidence rate has tripled to 17.8% (30). This might be attributed to the modern cattle management and feed treatment since the development of LAs are generally attributed to the transition to diets high in rapidly fermentable starch (28). Consumption of diets high in starch can lead to excessive production of SCFAs, lowering the ruminal pH, which can lead to ruminal acidosis. In turn, compromised rumen epithelial wall or any breach of the epithelial layer in the alimentary tract can allow causal microbes to enter the liver through the hepatic portal vein (31). It’s not clear if the diversity of liver microbial community is associated with the severity of liver abscess. And there are no early diagnostic tools available for the detection or prevention of LAs.

The study of microbial communities in the abscessed liver traditionally relied on culture-based methods, with which *Fusobacterium necrophorum* was reported as the primary causal agent for liver abscess (32). More recently, 16 s rRNA sequencing method has been used to profile the microbial community with the aim to assess the effectiveness of in-feed therapeutics in reducing liver abscess (33). Though LA and its associated microbial communities are typically perceived as a condition seen in diseased cattle, a recent study performed by Stotz and coauthors (34) reported the presence of an inherent microbial communities in the normal, non-abscessed bovine liver parenchyma, directly challenging the common assumption that liver, as an immunological organ, will not tolerant bacterial colonization within itself.

Calves fed a highly fermentable diet during the weaning period are the most vulnerable at developing ruminal acidosis. The increasing ruminal acidotic conditions resultant from the highly fermentable diet and induction of rumen wall damage at a critical window that coincides with substantial microbial ecological development could result in gut barrier dysfunction in the calf. This could drive microbial translocation and subsequent liver microbial colonization that leads to inflammation later in life. Using our established model of feed induced ruminal acidosis in young calves, we have observed significant changes in both rumen epithelial and liver transcriptomes (35, 36). Additionally, in the rumen epithelium and liver, we identified significant association between the host gene expression and the abundance of the microbial communities attached to the rumen epithelial. In this study, we dived into the microbial communities embedded in the liver tissue, which is a novel angel of the study and has not been reported before in young calves fed an acidosis-inducing or -blunting diet. Using a whole transcriptome sequencing technology, our experiment is designed to capture the active microbes in the liver. In turn, we investigated the abundance correlation between the microbial taxa in the liver and previously generated transcriptomic profiles in the host liver.

## 2. Materials and methods

### 2.1. Feed induced acidosis in young calves

Calves included in this study were part of a larger study that has been published (14, 35, 36). Animal protocol (A005848) is approved by the Animal Care and Use Committee at the University of Wisconsin-Madison. All the procedures relating to animal care and use in this study were implemented in accordance with the guidelines and regulations by the US Dairy Forage Research Center Farm.

The procedure for feed induced acidosis was described in our previous studies. In brief, 8 Holstein bull calves were enrolled for this experiment. For the first 8 weeks after birth, calves were housed in individual calf hutches (4.8 m^2^/calf), then they were moved to divided super hutches (5.0 m^2^/calf) through 16 weeks of age. Two diets were administered to the calves. One was a starch-rich, low-fiber diet. This diet was designed to cause ruminal acidosis (Aci; pelleted, 42.7% starch, 15.1% neutral detergent fiber (NDF), and 5.56% sugar). Texturized starter was fed as a control, which was designed to blunt ruminal acidosis (Con, 35.3% starch, 25.3% NDF, and 6.17% sugar). Complete nutrient composition of each diet was listed in companion manuscript (37). Four calves were randomly assigned to each diet treatment. The trial started at 1 week of age and lasted until 17 weeks. At 3 weeks of age, soft rubber cannulas (28 mm i.d.) were fitted to each calf following the method by Kristensen and coauthors (38). Between 7 and 9 weeks of age, larger soft rubber cannulas (51 mm i.d.; Bar-Diamond Inc., Parma, ID, United States) were used to replace the original cannulas due to accommodate the increase in the size of the fistula. A calibrated pH electrode was inserted into the rumen through the canula before each collection to measure rumen pH. A measured amount of starter was offered daily at 0800 h with refusals determined daily. Ruminal pH was tested at seven time-points (−8, −4, 0, 2, 4, 8, and 12 h relative to starter feeding) in a single day every other week from 6 wk to 16 wk. At 8 weeks, rumen epithelial biopsy was performed at the cranial sac using a uterine biopsy tool. At 17 weeks of age, all the calves were euthanized. Liver and rumen tissues were harvested. To ensure aseptic collection of the liver tissues, one leaf of liver was removed and rinsed with sterile 1X PBS. The surface of the liver prior to incision was further wiped with a sterile alcohol wipe. Then a surgery-grade scalpel was used to make an incision in the size of 5 cm and the liver tissue inside, beneath the incision was removed with a new set of sterile forceps and scalpel. On a sterile surface, the liver tissue was cut 4–5 mm^2^ pieces and placed into Eppendorf safe-lock tubes (Eppendorf North America, Hauppauge, NY, United States). Collected tissues were immediately frozen in liquid nitrogen and stored at −80°C for further RNA extraction.

### 2.2. RNA extraction and sequencing library preparation for liver tissues

For host liver RNA extraction, 50 mg of liver tissue was homogenized with Precellus Evolution instrument (Bertin Instrument, France) at 6800 rmp for 30 s per cycle and repeated 4 times. The tissue homogenate was put in ice between cycles for 1 min. After tissue homogenization, total RNA extraction was done using RNeasy mini Kit (Qiagen, Germany) following manufacturer’s instructions. Rumen papillae issues were homogenized on the Precyllus homogenizer (Bertin Instrument, France) at 7,500 RPM for 30 s per cycle, with 4 repeats. The tissue homogenate was place in ice for 1 min between cycles. After homogenization, total RNAs were extracted from both tissue types following the miRNeasy protocol with a QIAcube instrument (Qiagen, Germany). The quality of extracted RNAs was assessed using Bioanalyzer RNA 6000 nano kit (Agilent Technologies, United States). RNA samples with RNA integrity number (RIN) value ≥8 were pursued for RNA quantification using Qubit (ThermoFisher Scientific, United States). A total of eight, RNA samples (4 samples per treatment and they all passed the RIN threshold) were prepared into RNA-sequencing libraries using Illumina TruSeq ribo-zero Gold kit following manufacturer’s instructions. For each sample, 1 μg of total RNA was used for sequencing library preparation. Quantification of prepared libraries was performed using a Kapa quantification kit (KK4873, Kapa systems, Roche, Switzerland) with a QuantStudio 5 RT-qPCR instrument (Thermo Fisher, United States). Library concentration was calculated using a Kapa quantification kit (KK4873, Roche, Switzerland), following manufacturer’s instruction. Using the concentration generated by Kapa kit, sequencing pooling was prepared according to the calculation offered by the pooling calculator.^1^ Pooled libraries were initially sequenced using an Illumina nano 300-cycle kit. The pooling was normalized further to ensure equal depth of sequencing of all the libraries, according to the index ratios generated by the nano kit run. The finally normalized, pooled libraries were sequenced on the Illumina NextSeq 500 instrument, using a high-output 300-cycle cartridge to generate paired-end, 2 × 150 bp reads.

### 2.3. Bioinformatics analysis and taxa classification of microbial reads for the liver tissues

Sequencing data analysis followed the procedure described in our previously published work (35, 36). RNA sequencing raw reads were mapped to the cattle reference genome (ARS-UCD 1.2) using STAR (2.5.2b) (39). Differential gene expression analysis between AC and Control treatment was performed by the cuffdiff package in cufflinks2 (40). Cattle genome unmapped reads were considered of microbial origin. To further enrich microbial reads, SortMeRNA (version, 2.1b) (41) was used to map host genome unmapped reads to the reference rRNA databases provided by Silva (release 119) (42) and Rfam 11.0 (43). The enriched rRNA reads were used for bacterial taxonomic classification, using Kraken2 (v.2.0.8-beta) (44). Raw-read counts at phylum and genus levels as identified by Kraken2 were normalized by sequencing depth following the method previously published. Briefly, the total number of reads mapped to the given taxonomic level was divided by 1,000,000 to obtain the “per million factor”; (2) the total number of reads mapped to each specific given taxonomic level was divided by the “per million factor” to yield the normalized read count.

### 2.4. Liver microbial community analysis

The multivariate method mixMC implemented in the mixOmics R package (45) was used to identify associations between the liver microbiome and the groups AC x Control. For this analysis, only taxa with relative abundance >0.01% were considered (Supplementary Table S1). Then, was used the sparse partial least square discriminant analysis (sPLS-DA) (46) to identify the microbial signatures related to the AC and control groups. We selected the optimal number of components based on the averaged balanced classification error rate using the maximum distance over 10 repeats of an sPLS-DA model with 2 components. The lowest average balanced classification error rate after tuning the sPLS-DA model was used to choose the optimal number of variables. The top 10 discriminant genera contributing to component 1 in sPLS-DA were plotted.

### 2.5. Association analysis between host gene expression and microbial taxa abundance in the liver

For the host gene expression data, gene-level, raw read counts for the host liver, were previously published by our group (36). For the association analysis done here, we used normalized read count (Supplementary Table S1). Normalized read counts at genus level was used as the measurement for microbial abundance analysis (Supplementary Table S2). Pearson’s *r* correlation analysis between the host liver gene expression and its microbial genus level abundance was performed by using the cor function in R. The cutoff values of |*r*| > 0.7 and *p* < 0.001 were used to determine significant correlations. The normalized read count of 2 was used to determine the presence/expression of taxa. For the genes with significant association with microbial taxa, gene ontology (GO) and pathway analysis were performed by using DAVID (v2022q4) (47).

### 2.6. Microbial community comparison between the liver and rumen epithelium at 17 weeks of age

For this analysis, we included the rRNA raw reads of the microbial community data of rumen epithelium tissues collected at 17 weeks of age. The raw reads for rumen epithelium were published by our group previously with an NCBI SRA accession number of PRJNA948013 (35). For both tissue types, genus-level, raw read counts calculated by Kraken2 (v.2.0.8-beta) (44) were used to perform differential abundance analysis between treatment groups, AC vs. Control using DEseq2 (v 1.41.5) (48). Microbial genera showed significant differential abundance (*p* < 0.05 and fold-change >2) between the treatment groups were compared between liver and rumen epithelium tissues.

### 2.7. Proof-of-concept analysis of previously identified microbial taxa in cattle abscessed liver

For proof-of-concept study, we assess the presence of previously reported microbial taxa significantly associated with liver abscess. Among these, several microbial taxa have been considered as the causal agent of liver abscess in cattle. They include *Fusobacterium necrophorum* (31, 49), *Trueperella pyogenes*, and *Salmonella enterica* (50, 51). *F. necrophorum* gained significant attention due to its ubiquitous presence in culture-based studies (50, 52, 53). In a recently comprehensive study, Whitlow and coauthors (54) reported significant abundance of *Fusobacterium*, *Bacteroides*, and *Porphyromonas*. And the discriminant genera for Fusobacteria-dominated abscess communities as *Acinetobacter*, *Lactobacillus*, *Psdudomonas*, and *Psychrobacter*. For Bacteroidetes-dominated abscesses, the discriminant genera included *Atopobium*, *Campylobacter*, *Filifactor*, *Helocococcus*, *Parvimonas*, and *Trueperella*. We examined the presence and abundance of these taxa in our samples. Stotz and coauthors (34) investigated the liver microbial communities in 6 months old. The genera for the top 10 most abundant OTUs reported by Stotz and coauthors were also included in the proof-of-concept analysis. To account for the variation in sequencing depth, normalized read count for each taxon was used for this analysis. For each taxon, its presence is accessed for the 4 samples in each treatment group and the average read count (Mean ± S.E.) was calculated for each treatment group.

## 3. Results

### 3.1. Enrichment of microbial reads from the total RNA sequencing reads

For each liver sample, an average of 44.7 M ± 1.7 M raw reads were obtained. An average of 74% ± 2% of these reads were mapped to the cattle genome. Using our bioinformatics filtering strategy, an average of 1.7 M ± 0.17 M raw reads per sample were of microbial rRNA origin. Accounting for an average of 15% ± 1% of the non-cattle reads. Enriched microbial, rRNA reads were used for microbial taxa classification using Kraken. Majority of the reads were successfully classified, with an average classification rate of 97 ± 0.34% with high confidence. Microbial taxa at both genus and phylum levels were followed for comparison analysis between liver and rumen tissues.

### 3.2. Liver microbial community and its changes between treatment groups at 17 weeks of age

**Figure 1:**
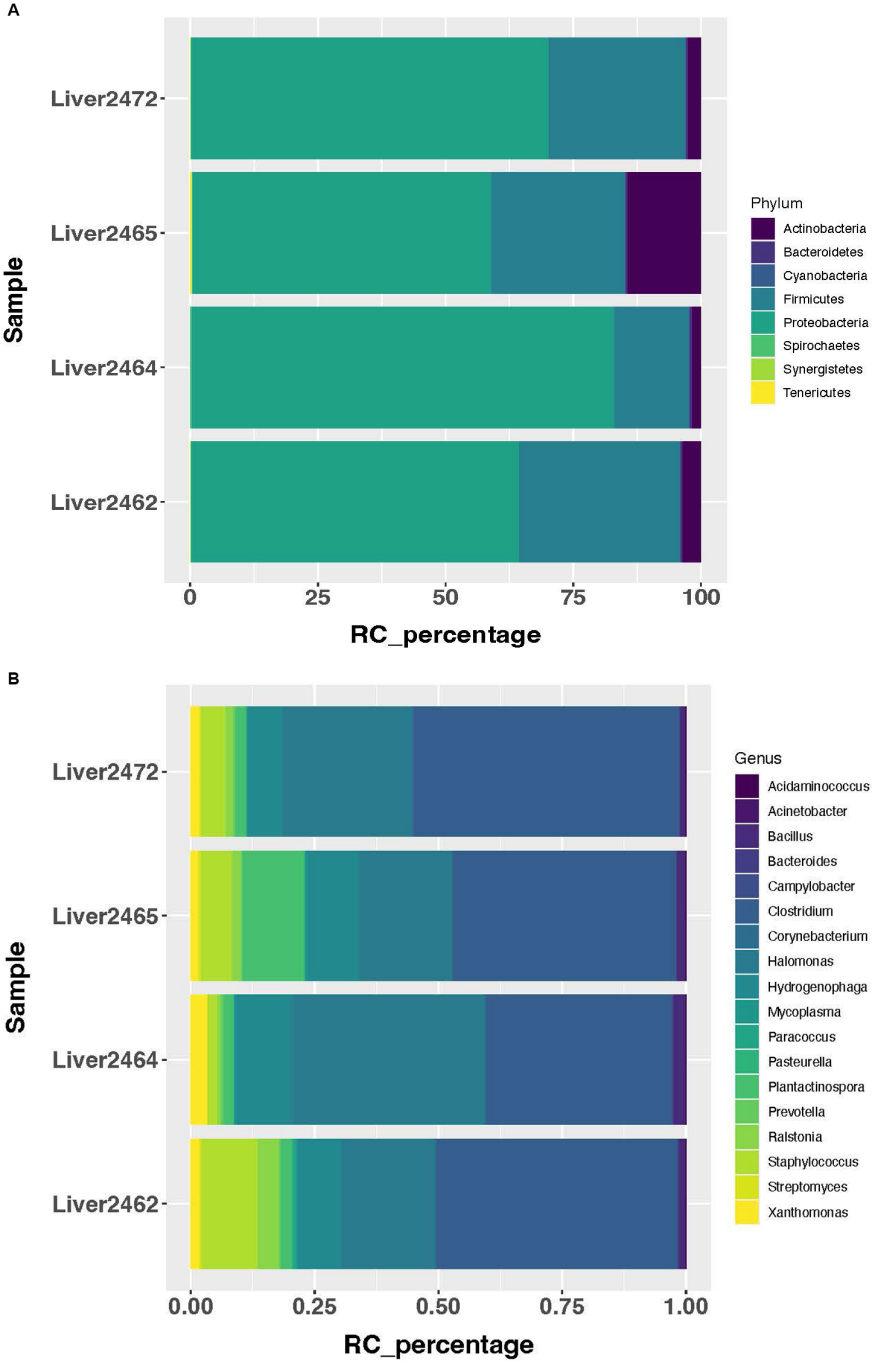
Most abundant microbial taxa identified in the treated group. **(A)** Most abundant microbial phyla in the treated group. **(B)** Most abundant microbial genera in the treated group.

**Figure 2:**
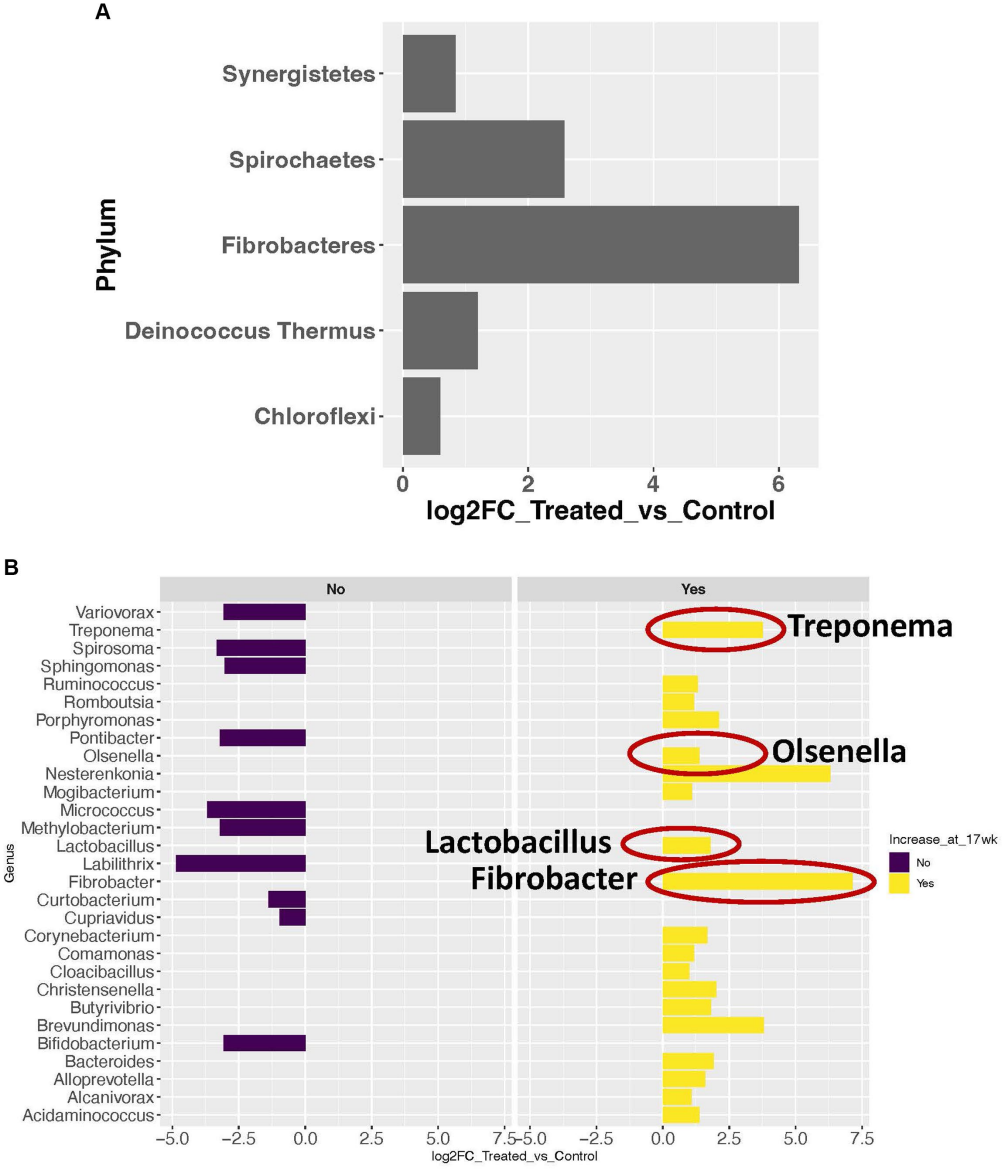
Microbial taxa with significant abundance changes between treated and control groups. **(A)** Significance increase in abundance in 5 phyla. **(B)** Significant abundance changes at the genus level. Dark purple bars represent the genera with significant decrease in abundance in the treated group. Yellow bars represent the genera with significant increase in abundance in the treated group. The genera with a red circle are the ones previously reported in abscessed liver in cattle.

Using a cutoff of average genus level read count (RC) of 5, 92 genera were determined as present in the liver in this study. A consistent microbial community profile was observed in the liver in treated group. In this group, more than 98% of the reads belong to 8 phyla, with Proteobacteria, Firmicutes, and Actinobacteria as the top 3 most abundant phyla (Figure 1A). And more than 95% of the reads belong to 18 genera (Figure 1B). At the phylum level, 5 phyla showed significant increase in transcript abundance in the treated group (Figure 2A). At the genus level, a total of 29 genera showed significant (*p* < 0.05) abundance changes (>2-fold) between the treatments (Figure 2B). Among these, 19 showed increases in the treated group, and 10 of them showed decrease in the treated group. *Fibrobacter*, *Treponema*, *Lactobacillus*, and *Olsenella* have been previously reported in abscessed liver in cattle.

**Figure 3:**
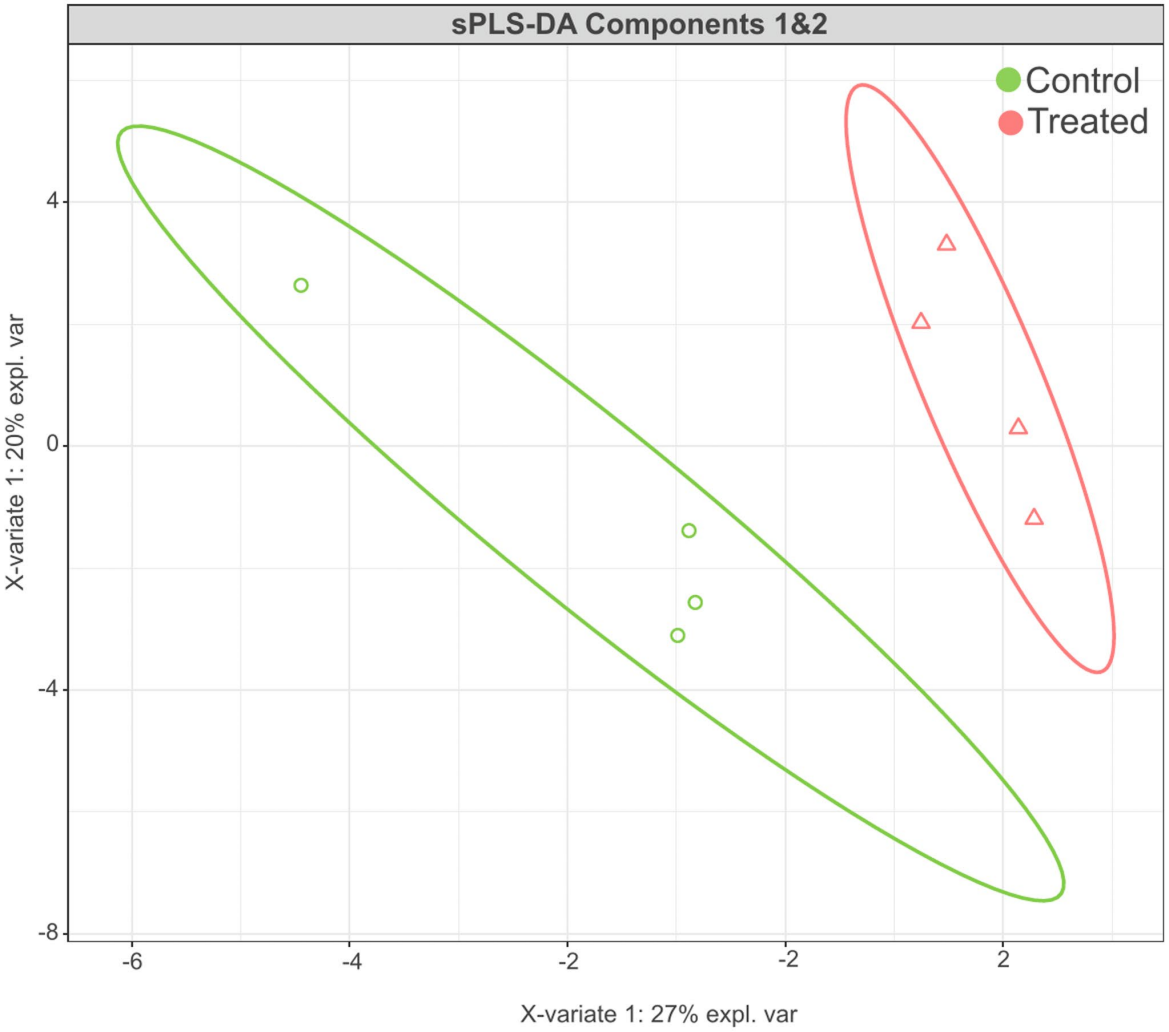
Results of sPLS-DA for microbial community profile at genus level in the liver of control and treated groups. Score plot of the first two components, with 95% confidence level.

**Figure 4:**
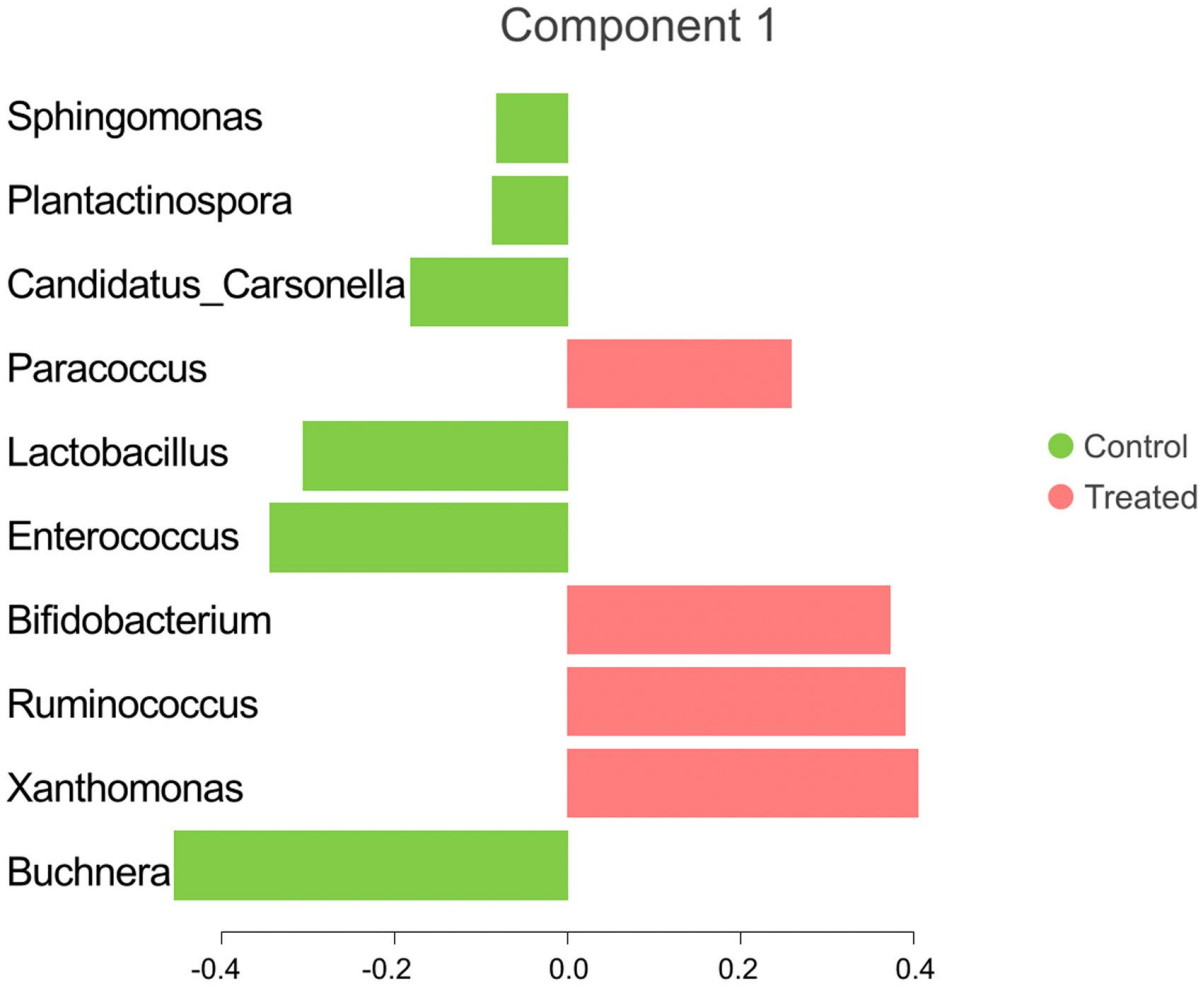
Microbial taxa contribution ranked from bottom (most important) to top. The colors indicate the treatment group in which the feature was most relevant.

The sPLS-DA analysis revealed a clear separation in the microbial community structure for the control vs. AC group (Figure 3). To identify the discriminant taxa for the AC and control group, sPLS-DA analysis estimated that 60% of the microbial signature selected in component 1 of the sPLS-DA characterized the liver microbiome of animals from the control group, which included *Buchnera*, *Enterococcus*, *Lactobacillus*, *Candidatus Carsonella*, *Plantactinospora*, and *Sphingomonas*. On the other hand, the microbial signature characterized the liver microbiome of the treated group comprised of *Xanthomonas*, *Ruminococcus*, *Bifidobacterium*, and *Paracoccus* (Figure 4).

### 3.3. The association analysis between host genes and microbial taxa

This association analysis was done using previously published, normalized, gene-level read count of host liver genes, and genus-level, normalized read count of the liver microbial community, which was obtained for this report. Using a cutoff of values of |*r*| > 0.7 and *p* < 0.001, 139 liver genes were identified with significant association with 38 microbial genera identified in the liver. The average normalized read counts of these genes were 159.97 ± 34.02 (Mean ± S.E.). The top 4 most highly expressed genes are *ITIH4*, *C4A*, *SPP2*, and *TMBIM6*. Their associated microbial genera are listed in Table 1. The genes with 4 or more associated microbial genera included *ARRDC3*, *TTC7A*, *MOSPD1*, *FAM105B*, *CTNNAL1*, *CRELD2*, *GMPPA*, *NUCB2*, and *CSAD* (Table 2). The microbial genera associated with 15 or more genes included *Megasphaera*, *Ruminococcus*, *Dialister*, *Treponema*, *Fibrobacter*, and *Prevotella*. GO analysis using the identified genes determined the enrichment in the following pathways: GO:006090 (pyruvate metabolic process); GO:004736 (pyruvate carboxylase activity). bta04918 (thyroid hormone synthesis). bta00510 (N-Glycan biosynthesis). GO:0003756 (protein disulfide isomerase activity); GO:0005788 (endoplasmic reticulum lumen); GO:0042470 (melanosome); GO:0005788 (endoplasmic reticulum lumen) and bta04141(Protein processing in endoplasmic reticulum); bta00510(N-Glycan biosynthesis); GO:0016021 (integral component of membrane) (Table 3).

**Table 1:** Most highly expressed genes and their associated microbial genera on treated and control samples.

Genus name	Associated microbial genera	Mean normalized RC of host gene	Mean normalized RC of microbial genera	Pearson’s *R*	*p*-value
*ITIH4*	Dialister	3,477	21.25	0.93	<0.001
*C4A*	Campylobacter	2043	75.75	0.96	<0.001
*SPP2*	Ralstonia, Staphylococcus	1829	1,612	0.95	<0.001
*TMBIM6*	Corynebacterium	1,510	28.5	0.95	<0.001

**Table 2:** Host liver genes identified with significant association with its embedded microbes.

Gene name	Associated microbial genera
*ARRDC3*	Blattabacterium, Buchnera, Candidatus_Carsonella, Escherichia, Mesorhizobium, Phocaeicola, Plantactinospora, Stenotrophomonas
*TTC7A*	Blattabacterium, Buchnera, Candidatus_Carsonella, Escherichia, Mycobacterium, Phocaeicola, Plantactinospora, Stenotrophomonas
*MOSPD1*	Buchnera, Candidatus_Carsonella, Escherichia, Mycobacterium, Plantactinospora, Stenotrophomonas
*FAM105B*	Buchnera, Escherichia, Mycobacterium, Plantactinospora, Stenotrophomonas
*CTNNAL1*	Blattabacterium, Candidatus_Carsonella, Mesorhizobium, Plantactinospora
*CRELD2*	Dialister, Fibrobacter, Ruminococcus, Treponema
*GMPPA*	Dialister, Fibrobacter, Ruminococcus, Treponema
*NUCB2*	Dialister, Fibrobacter, Ruminococcus, Treponema
*CSAD*	Escherichia, Mycobacterium, Plantactinospora, Stenotrophomonas

**Table 3:** Liver microbial taxa identified with significant association with host liver genes.

Genus	Associated host genes	GO terms of associated genes
*Megasphaera*	PC, SSR2TMED9, HNRNPM, SEC16A, COPG1, DNAJA3, CLPTM1L, SLC25A47, SHC1, PLBD2, SLC39A7, ALKBH5, FKBP11, GALE, CNPY2, VARS, CNPY3, UBL7, FBXL14, KLHL25, MAP2K2, SEC13, TRABD, CAD, DGKZ, ATXN7L3, TRAP1, NF2, RUVBL1	GO:006090 (pyruvate metabolic process); GO:004736 (pyruvate carboxylase activity)
*Ruminococcus*	HSP90B1, SERPINA3-3, HSPA5, PDIA4, SEL1L, UGGT1, DNAJC3, SPCS2, EDEM1, HYOU1, ERP44, ANKH, HM13, SERP1, MANF, CKAP4, SEC23B, SDS, MOGS, SLC35E1, CRELD2, DNAJB11, NUCB2, ALG5, UNC13B, RCN2, GMPPA	bta04918 (thyroid hormone synthesis)
*Dialister*	ITIH4, MST1, HDLBP, TMEM66, RPN1, SND1, SEC61A1, SEC16A, HM13, MAN1B1, MOGS, TOLLIP, PRDX4, CRELD2, NUCB2, GPRC5C, TRABD, TXNDC5, GMPPA	bta00510 (N-Glycan biosynthesis)
*Treponema*	HSP90B1, CALR, PDIA4, PDIA3, PDIA6, SEL1L, UGGT1, DNAJC3, SPCS2, HYOU1, ERP44, TMEM214, GOLPH3L, CRELD2, DNAJB11, NUCB2, RCN2, GMPPA	GO:0003756 (protein disulfide isomerase activity); GO:0005788 (endoplasmic reticulum lumen)
*Fibrobacter*	HSP90B1, CALR, PDIA4, PDIA3, PDIA6, SEL1L, SPCS2, HYOU1, ERP44, TMEM214, GOLPH3L, CRELD2, DNAJB11, NUCB2, RCN2, GMPPA	GO:0042470 (melanosome); GO:0005788 (endoplasmic reticulum lumen)
*Prevotella*	HDLBP, RPN1, SND1, SEC61A1, ERP29, ANKH, HM13, MAN1B1, TOLLIP, FKBP2, PRDX4, VARS, OSBP, TRABD, CAD	bta04141(Protein processing in endoplasmic reticulum); bta00510(N-Glycan biosynthesis); GO:0016021 (integral component of membrane)

### 3.4. Microbial community comparison between the liver and rumen epithelium at 17 weeks of age

**Figure 5:**
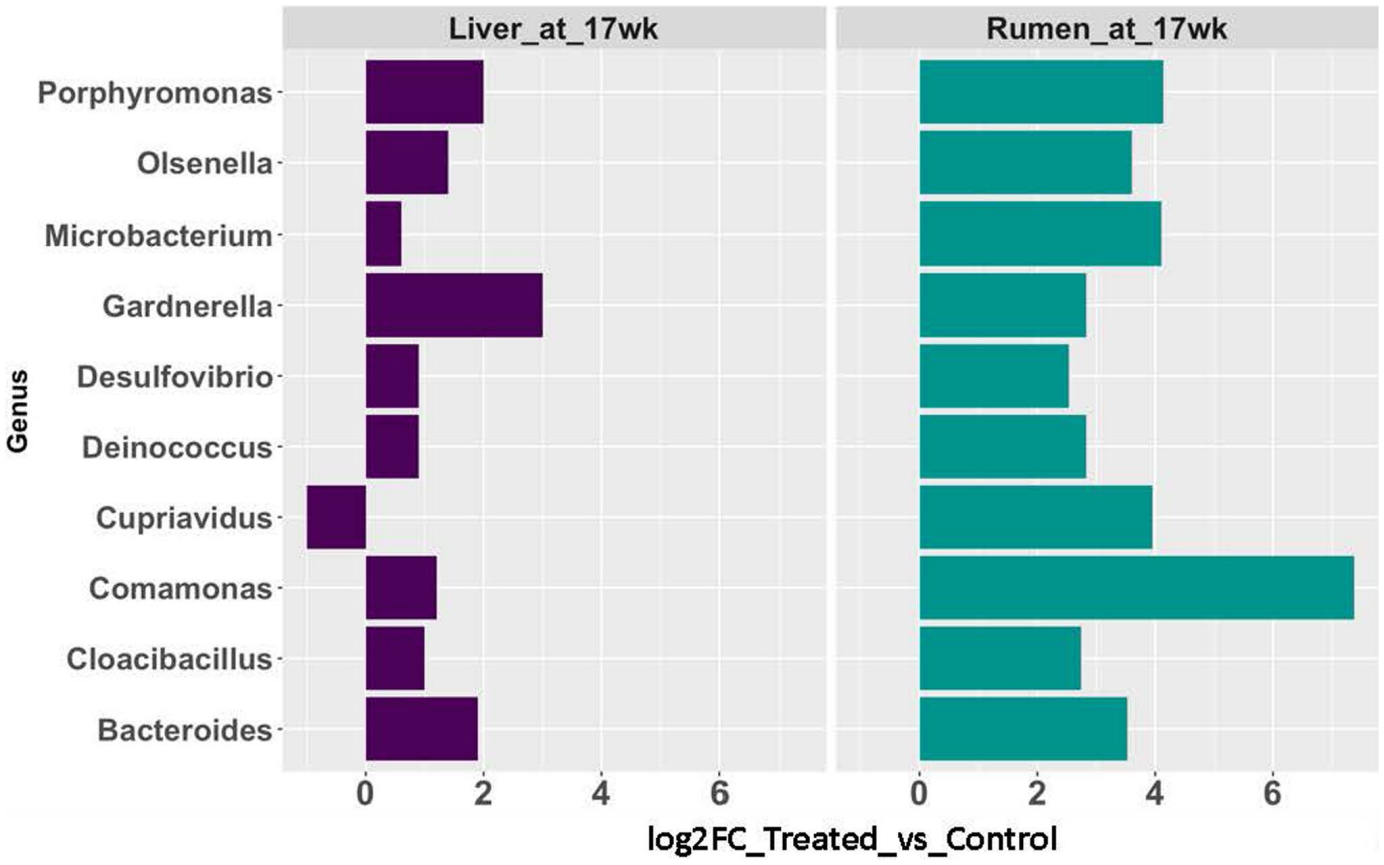
Abundance changes of microbial taxa identified in both rumen epithelial (teal blue bars) and liver (purple bars) at 17 weeks of age.

The rumen epithelium tissues collected at 17 weeks of age were included in this comparison. In the rumen epithelium collected at 17 weeks of age, 57 genera showed significant changes in rRNA transcript abundance (*p* < 0.05 and fold-change >2). Nine of the genera were also identified with a significant increase (*p* < 0.05 and fold-change >2) in abundance in the AC group in liver tissues (Figure 5).

### 3.5. Identification of microbial genera previously identified as discriminant or highly abundant in abscessed liver

For the proof-of-concept analysis, 17 microbial genera previously reported either as discriminant taxa for abscessed liver in cattle or as highly abundant in the abscessed liver were included in the analysis. A predominant majority (15 out 17) of them is detected in our study (Table 4). Several of these taxa showed differential abundance between control and treated groups. Across all the samples included in this study, the average RC of these genera ranged from 4.75 to 2,128. In both groups, the most abundant genera (RC > 200) include *Prevotella*, *Acinetobacter*, *Bacteroides*, *Campylobacter*, *Acinetobacter*, *Pseudomonas*, *Lactobacillus*, and *Campylobacter*. The genera with lower abundance (RC < 100) include *Fusobacterium*, *Salmonella*, and *Porphyromonas*. The rest of the genera have no detectable read counts, or they were only detected in one of the samples in either treatment group.

**Table 4:** Average normalized read-counts for the genera previously reported as highly abundant in liver abscesses, comparing treated and control samples.

Genus name	Mean read count	
	In treated samples	In control samples
*Acinetobacter*	573.75 ± 101.3	655 ± 326.05
*Bacteroides*	468.25 ± 123.24	128.5 ± 40.45
*Campylobacter*	916 ± 249.03	603.25 ± 170.86
*Fusobacterium*	68.5 ± 25.86	54.75 ± 11.7
*Trueperella*	(12)*	(9)*
*Salmonella*	83.25 ± 17.03	86 ± 20.31
*Porphyromonas*	82 ± 31.39	21 ± 13.07
*Prevotella*	2,128 ± 857	1,654 ± 461
*Acinetobacter*	573.75 ± 101.33	655.25 ± 326.05
*Lactobacillus*	298.5 ± 158.31	74 ± 19.56
*Pseudomonas*	652.25 ± 145.95	624.25 ± 283.02
*Psychrobacter*	5.75 ± 3.32	16.5 ± 4.29
*Atopobium*	Not present	Not present
*Filifactor*	(28)*	Not present
*Helocococcus*	Not present	Not present
*Parvimonas*	11.25 ± 5.17	4.75 ± 2.75

^*^The genus is detected in only one sample, and normalized read count in the only sample is listed in parentheses.

## 4. Discussions

### 4.1. Host liver genes with significant association with its microbial taxa

Through the association analysis of host gene expression level and microbial taxa abundance in the liver, several valuable insights surfaced. Four highly expressed genes were identified with significant association (Pearson’s *r* > 0.9 and *p* < 0.0001). Two of them (*ITIH4* and *C4A*) are involved in inflammatory response as previously reported. *ITIH4* was reported as an inflammatory biomarker in bacterial bloodstream infection (55), in addition. To its association with inflammatory bowel disease (56). This gene encodes an acute phase protein and is primarily expressed in liver. Its significant increase in the serum during acute phases have been reported in human patients (57). In bovine, several acute phase proteins are routinely used as inflammatory markers. They include serum amyloid A, haptoglobin, alpha-1-acid glycoprotein, and alpha-1-proteinase inhibitor (58–60). And as a newly established acute phase protein, the serum concentration of ITIH4 protein positively correlated with the disease severity of mastitis and its increased concentration was also confirmed in bovine with bovine respiratory syncytial virus infection (61). Interestingly, the microbial taxa associated with this gene is *Dialister*, which was reported as the causal pathogen in human liver abscesses (62). Though the causal relationship between *Dialister* and liver abscess in cattle has not been established, *ITIH4* stands as a high potential biomarker to be developed for the early detection of bacterial induced liver or blood inflammation in cattle.

For the association analysis between the expression of liver genes and the microbial taxa abundance in the liver, interesting insights were revealed by the function of the genes and the GO pathways they were involved with. For the genes associated with the highest number of microbial taxa, they had previously reported roles in energy and lipid metabolism, and regulation of inflammation in the intestine and liver. These findings suggest that at the host physiology level, significant responses driven by microbial community changes are already underway before the overt expression of clinical signs of ruminal acidosis. For the identified GO terms, our analysis indicated that liver microbial community may contribute significantly to liver physiology changes resultant from feed induced acidosis. This is reflected by the enrichment of cellular component (GO:0005788, endoplasmic reticulum lumen; GO:0016021, integral component of membrane), pyruvate metabolism (GO:006090, pyruvate metabolic process; GO:004736, pyruvate carboxylase activity). Both enrichments were previously identified for the differentially expressed genes in the liver between the treatment groups (36). Specifically, pyruvate was reported as a correction buffer to intracellular acidosis (63). Furthermore, an enrichment of thyroid hormone synthesis pathway was identified for the genes associated with the tissue-embedded microbes. Low thyroid hormone, T3, was reported as a predictor of poor prognosis in patients with pyogenic liver abscess (64). Liver is important for the excretion, transport, metabolism and storage of thyroid hormones (65), thought currently studies of the abundance of thyroid hormone in cattle primarily primarily focused on pregnancy and lactation (66, 67). Our data suggested that liver microbial community may impact thyroid hormone profile in the liver in the event of liver metabolic acidosis caused by feed induced ruminal acidosis.

### 4.2. Concurrent changes in both the rumen epithelial and liver microbial community changes

We observed concurrent changes in the microbial communities in both the liver and rumen epithelial at 17 weeks of age, evidenced by the 10 genera with significant abundance changes between the AC and control groups. Nine of these genera showed the same trend of expression changes in both tissue types. In previous report by our group, significant lesions were identified in the rumen epithelial tissues at 17 weeks of age (14). Combined with our observation, it is possible that compromised tight junction and gut barrier function may take place earlier than 17 weeks of age. Once compromised, the damaged gut barrier opens the door for the translocation of microbes from the rumen to the liver via the circulatory system. Our findings provided insight into host rumen transcriptome and associated microbial community changes in both the rumen and liver resultant from prolonged acidosis in post weaning calves. The concurrent shifts in microbial genus abundance in both the liver and rumen at 17 weeks of age indicated the potential translocation of microbes from the rumen epithelial to the liver. Given the important role of the liver as a metabolic organ, our findings warrant future investigation into the role of liver microbiome in the development and host response to ruminal acidosis.

### 4.3. Significant presence of previously identified microbial taxa and new taxa in the liver of calves with feed-induced acidosis

As a proof-of-concept investigation, we checked the rRNA transcript abundance of 17 microbial genera previously reported as discriminant or highly abundant taxa for abscessed liver. Majority of them were detected in our study. *Fusobacteria* has long been considered the primary causal pathogen to liver abscess in cattle since this microbial taxon has been consistently identified using culture-based methods (50, 52, 53, 68) or 16 s rRNA amplicon-based DNA sequencing-based method (33). However, the limitation of each of these methods may lead to biased assessment. In our study, the transcript presence is substantial for this taxon as identified by our RNA sequencing-based method, though it does not appear to be the most abundant as measured by the read count of rRNA transcripts. To be successfully identified by culture-based methods, the culture media must be compatible to the targeted microbes. Additionally, anaerobic microbes require an environment void of oxygen, making it difficult to grow anaerobic microbes in a lab setting. DNA based sequencing cannot differentiate dead or lysed cells from the live cells. Despite its predominant abundance identified in the 16 s rRNA amplicon-based studies, it does not directly indicate that this genus is actively transcribed. And because of this limitation, many microbial genera determined as highly abundant by DNA-based sequencing may suffer the noise coming from the DNA extracted from dead cells.

In addition to the successful identification of many previously identified microbial genera in abscessed liver, many new microbial genera were detected in our study with significant rRNA transcript abundance by our transcriptome sequencing approach. Interestingly, several of the new genera identified in our study have already been reported as potential causal pathogens in human liver abscess and other high impact diseases in livestock. For example, the genera *Pasteurella* and *Clostridium* presented a high abundance in the liver samples in our study (Supplementary Table S2). *Pasteurella* sp. was previously reported in liver, lung and spinal epidural abscess in human (69, 70). *Clostridium* sp. (71, 72), and *Pasteurella* sp. (69) were reported in human and domestic animal liver abscess. *Clostridium* is an anaerobic spore-forming bacteria. Its presence is often found in the soil, feedstuff or manurer (73). Once ingested, the *Clostridium* can proliferate in the intestinal mucosa, reach the liver via the circulatory system, and persist in the phagocytic cells (74). When the animal’s diet change or experiences tissue injury, it creates a favorable condition for the bacteria to proliferate and become pathogenic causing infection in the liver and other organs (74). *Pasteurella* sp. is highly prevalent in domestic and wild animal populations (75). The symptomatic infection with *Pasteurella* was considered a high-impact disease in livestock, according to World Animal Health Organization.^2^ Though not all the new microbial taxa identified in our study showed significant changes between the treatment groups, this new repertoire of actively transcribed microbial taxa identified in our study paves the way for future in depth analysis of the liver microbiome and its connection with feed induced acidosis. Of note, the timing of the sample collection is worth additional efforts since microbial transcripts might not be produced at a constant rate and a more expansive study that covers multiple time points might help uncover novel causal microbes to liver abscess in cattle.

Our study and others indicate that the pathogenesis of liver abscess is polymicrobial. The pathobiology of many of the newly identified taxa in our study remains to be elucidated. Additionally, majority of previously published work focus on adult cattle. Our study is one of the few that focuses on young calves. Most importantly, our study suggested that, to capture meaningful changes in the liver microbial community, transcriptome-based sequencing may offer a new layer of insights by its discriminating power of capturing actively transcribed transcripts from liver embedded microbial cells.

## 5. Conclusion

This study investigated the tissue-embedded microbial community in the liver in calves with or without feed induced acidosis. Many of the previously reported microbial taxa in cattle abscessed were identified in this study. Most importantly, by employing metatranscriptome sequencing, our study provided a glimpse of many new microbes that showed significant abundance changes between treatment groups. The association analysis between host liver gene expression and its microbial community abundance suggested that liver microbiome may play a critical role in host liver physiology. Specifically, genes involved in thyroid hormone related pathway were identified with significant association with the microbial taxa in the liver, though thyroid hormone responses were previously only investigated in cattle during pregnancy or lactation. These novel findings emphasize the need for further in-depth analysis, which will help paint a full picture of the diversity and assembly of tissue embedded microbes in the liver, and their physiological roles in metabolic acidosis in the liver resultant from feed-related ruminal acidosis.

## Data availability statement

The datasets presented in this study can be found in online repositories. The names of the repository/repositories and accession number(s) can be found at: https://www.ncbi.nlm.nih.gov/bioproject; PRJNA948013, PRJNA962842.

## Ethics statement

The animal study was approved by The Animal Care and Use Committee at the University of Wisconsin-Madison. The study was conducted in accordance with the local legislation and institutional requirements.

## Author contributions

WL conceived the RNA sequencing experiment and analyzed the host and microbial transcriptome sequencing data. AL and BM processed the raw tissues, extracted total RNA, performed RNA quality check, and RNA sequencing library preparation. PF performed microbial signature analysis. WL and PF contributed to the writing of the manuscript. All authors contributed to the article and approved the submitted version.

## Footnotes

^1^https://support.illumina.com/help/pooling-calculator/pooling-calculator.htm

^2^www.oie.int

## References

[1] Khan MA Bach A Weary DM von Keyserlingk MAG. Invited review: transitioning from milk to solid feed in dairy heifers. J Dairy Sci. (2016) 99:885–902. doi: 10.3168/jds.2015-9975, PMID: 26709160

[2] Khan MA Weary DM von Keyserlingk MA. Invited review: effects of milk ration on solid feed intake, weaning, and performance in dairy heifers. J Dairy Sci. (2011) 94:1071–81. doi: 10.3168/jds.2010-3733, PMID: 21338773

[3] Guzman CE Bereza-Malcolm LT De Groef B Franks AE. Presence of selected methanogens, fibrolytic bacteria, and proteobacteria in the gastrointestinal tract of neonatal dairy calves from birth to 72 hours. PLoS One. (2015) 10:133048. doi: 10.1371/journal.pone.0133048, PMID: 26186002 PMC4505879

[4] Jami E Israel A Kotser A Mizrahi I. Exploring the bovine rumen bacterial community from birth to adulthood. ISME J. (2013) 7:1069–79. doi: 10.1038/ismej.2013.2, PMID: 23426008 PMC3660679

[5] Furman O Shenhav L Sasson G Kokou F Honig H Jacoby S. Stochasticity constrained by deterministic effects of diet and age drive rumen microbiome assembly dynamics. Nat Commun. (2020) 11:1904. 32312972 10.1038/s41467-020-15652-8 PMC7170844

[6] Cersosimo LM Radloff W Zanton GI. Microbial inoculum composition and pre-weaned dairy calf age Alter the developing rumen microbial environment. Front Microbiol. (2019) 10:1651. doi: 10.3389/fmicb.2019.01651 31396179 PMC6664089

[7] Park T Cersosimo LM Li W Radloff W Zanton GI. Pre-weaning ruminal administration of differentially-enriched, rumen-derived inocula shaped rumen bacterial communities and co-occurrence networks of post-weaned dairy calves. Front Microbiol. (2021) 12:625488. doi: 10.3389/fmicb.2021.625488, PMID: 33717013 PMC7952535

[8] Li W Edwards A Riehle C Cox MS Raabis S Skarlupka JH. Transcriptomics analysis of host liver and meta-transcriptome analysis of rumen epimural microbial community in young calves treated with artificial dosing of rumen content from adult donor cow. Sci Rep. (2019) 9:790. doi: 10.1038/s41598-018-37033-4 30692556 PMC6349911

[9] Meale SJ Popova M Saro C Martin C Bernard A Lagree M. Early life dietary intervention in dairy calves results in a long-term reduction in methane emissions. Sci Rep. (2021) 11:3003. doi: 10.1038/s41598-021-82084-9 33542279 PMC7862406

[10] Dias J Marcondes MI Motta de Souza S Cardoso da Mata ESB Fontes Noronha M Tassinari Resende R. Bacterial community dynamics across the gastrointestinal tracts of dairy calves during preweaning development. Appl Environ Microbiol. (2018) 84:17. doi: 10.1128/AEM.02675-17, PMID: 29475865 PMC5930334

[11] Dill-McFarland KA Weimer PJ Breaker JD Suen G. Diet influences early microbiota development in dairy calves without long-term impacts on milk production. Appl Environ Microbiol. (2019) 85:18. doi: 10.1128/AEM.02141-18, PMID: 30367001 PMC6328763

[12] Kehoe SI Dill-McFarland KA Breaker JD Suen G. Effects of corn silage inclusion in preweaning calf diets. J Dairy Sci. (2019) 102:4131–7. doi: 10.3168/jds.2018-15799, PMID: 30879818

[13] Flatt WP Warner RG Loosli JK. Influence of purified materials on the development of the ruminant stomach. J Dairy Sci. (1958) 302:91138. doi: 10.3168/jds.S0022-0302(58)91138-X, PMID: 36965544

[14] Gelsinger SL Coblentz WK Zanton GI Ogden RK Akins MS. Physiological effects of starter-induced ruminal acidosis in calves before, during, and after weaning. J Dairy Sci. (2020) 103:2762–72. doi: 10.3168/jds.2019-17494, PMID: 31882217

[15] Chishti GA Salfer IJ Suarez-Mena FX Harvatine KJ Heinrichs AJ. Short communication: relationships between physical form of oats in starter, rumen pH, and volatile fatty acids on hepatic expression of genes involved in metabolism and inflammation in dairy calves. J Dairy Sci. (2020) 103:439–46. doi: 10.3168/jds.2019-16296, PMID: 31733869

[16] Bull LS Bush LJ Friend JD Harris B Jr Jones EW. Incidence of ruminal parakeratosis in calves fed different rations and its relation to volatile fatty acid absorption. J Dairy Sci. (1965) 48:1459–66. doi: 10.3168/jds.S0022-0302(65)88499-5, PMID: 5864521

[17] Wood KM Palmer SI Steele MA Metcalf JA Penner GB. The influence of age and weaning on permeability of the gastrointestinal tract in Holstein bull calves. J Dairy Sci. (2015) 98:7226–37. doi: 10.3168/jds.2015-9393, PMID: 26278496

[18] Kirschner N Houdek P Fromm M Moll I Brandner JM. Tight junctions form a barrier in human epidermis. Eur J Cell Biol. (2010) 89:839–42. doi: 10.1016/j.ejcb.2010.07.010, PMID: 20732726

[19] Gatt M Reddy BS Mac Fie J. Review article: bacterial translocation in the critically ill - evidence and methods of prevention. Aliment Pharm Ther. (2007) 25:741–57. doi: 10.1111/j.1365-2036.2006.03174.x 17373913

[20] Sanders DS. Mucosal integrity and barrier function in the pathogenesis of early lesions in Crohn's disease. J Clin Pathol. (2005) 58:568–72. doi: 10.1136/jcp.2004.021840, PMID: 15917403 PMC1770702

[21] Anderson RC Cookson AL McNabb WC Park Z McCann MJ Kelly WJ. Lactobacillus plantarum MB452 enhances the function of the intestinal barrier by increasing the expression levels of genes involved in tight junction formation. BMC Microbiol. (2010) 10:316. doi: 10.1186/1471-2180-10-316, PMID: 21143932 PMC3004893

[22] Fasana CF Donelli G Uzzau S Kaper JB Margaretten K Ding X. Zonula occludens toxin modulates tight junctions through protein kinase C-dependent actin reorganization, in vitro. J Clin Invest. (1995). doi: 10.1172/JCI118114 PMC185254 7635964

[23] Ewaschuk JB Diaz H Meddings L Diederichs B Dmytrash A Backer J. Secreted bioactive factors from Bifidobacterium infantis enhance epithelial cell barrier function. Am J Physiol Gastrointest Liver Physiol. (2008) 295:G1025–34. doi: 10.1152/ajpgi.90227.2008, PMID: 18787064

[24] Bansal T Alaniz RC Wood TK Jayaraman A. The bacterial signal indole increases epithelial-cell tight-junction resistance and attenuates indicators of inflammation. Proc Natl Acad Sci U S A. (2010) 107:228–33. doi: 10.1073/pnas.0906112107, PMID: 19966295 PMC2806735

[25] Hamer HM Jonkers D Venema K Vanhoutvin S Troost FJ Brummer RJ. Review article: the role of butyrate on colonic function. Aliment Pharmacol Ther. (2008) 27:104–19. doi: 10.1111/j.1365-2036.2007.03562.x, PMID: 17973645

[26] Shinji Fukuda HT Hase K Oshima K Nakanishi Y Yoshimura K Tobe T. Bifidobacteria can protect from enteropathogenic infection through production of acetate. Nature. (2011) 469:543–7. doi: 10.1038/nature09646, PMID: 21270894

[27] Brown TR Lawrence TE. Association of liver abnormalities with carcass grading performance and value. J Anim Sci. (2010) 88:4037–43. doi: 10.2527/jas.2010-3219 20817855

[28] Reinhardt C Hubbert M. Control of liver abscesses in feedlot cattle: a review. Prof Anim Sci. (2015) 31:101–8. doi: 10.15232/pas.2014-01364

[29] Smith H. Beef liver condemnations. J Anim Sci. (1940) 1940:272–6.

[30] Eastwood LC Boykin CA Harris MK Arnold AN Hale DS Kerth CR. National Beef Quality Audit-2016: Transportation, mobility, and harvest-floor assessments of targeted characteristics that affect quality and value of cattle, carcasses, and by-products. Trans. Anim. Sci. (2017) 1:229–38. doi: 10.2527/tas2017.0029 PMC7250433 32704647

[31] Nagaraja TG Narayanan SK Stewart GC Chengappa MM. Fusobacterium necrophorum infections in animals: pathogenesis and pathogenic mechanisms. Anaerobe. (2005) 11:239–46. doi: 10.1016/j.anaerobe.2005.01.007, PMID: 16701574

[32] Nagaraja TG Chengappa MM. Liver abscesses in feedlot cattle: a review. J Anim Sci. (1998) 76:287–98. doi: 10.2527/1998.761287x, PMID: 9464910

[33] Amachawadi RG Tom WA Hays MP Fernando SC Hardwidge PR Nagaraja TG. Bacterial community analysis of purulent material from liver abscesses of crossbred cattle and Holstein steers fed finishing diets with or without tylosin. J Anim Sci. (2021) 99:76. doi: 10.1093/jas/skab076, PMID: 33693672 PMC8075120

[34] Stotz MK Henry DD Crossland WL. Characterization of bacterial DNA identified in abscessed and non-abscessed bovine hepatic tissue at the time of harvest. J Anim Sci. (2021) 99:280. doi: 10.1093/jas/skab280, PMID: 34610106 PMC8525596

[35] Li W Gelsinger S Edwards A Riehle C Koch D. Transcriptome analysis of rumen epithelium and meta-transcriptome analysis of rumen epimural microbial community in young calves with feed induced acidosis. Sci Rep. (2019) 9:4744. doi: 10.1038/s41598-019-40375-2 30894588 PMC6426933

[36] Li W Gelsinger S Edwards A Riehle C Koch D. Changes in meta-transcriptome of rumen epimural microbial community and liver transcriptome in young calves with feed induced acidosis. Sci Rep. (2019) 9:18967. doi: 10.1038/s41598-019-54055-8 31831817 PMC6908691

[37] Gelsinger SL Coblentz WK Zanton GI Ogden RK Akins MS. Ruminal in situ disappearance and whole-tract digestion of starter feeds in calves before, during, and after weaning. J Dairy Sci. (2019) 102:2196–206. doi: 10.3168/jds.2018-15551, PMID: 30639014

[38] Kristensen NB Engbaek M Vestergaard M Harmon DL. Technical note: ruminal cannulation technique in young Holstein calves: effects of cannulation on feed intake, body weight gain, and ruminal development at six weeks of age. J Dairy Sci. (2010) 93:737–42. doi: 10.3168/jds.2009-2488, PMID: 20105545

[39] Dobin A Davis CA Schlesinger F Drenkow J Zaleski C Jha S. STAR: ultrafast universal RNA-seq aligner. Bioinformatics. (2013) 29:15–21. doi: 10.1093/bioinformatics/bts635, PMID: 23104886 PMC3530905

[40] Trapnell C Roberts A Goff L Pertea G Kim D Kelley DR. Differential gene and transcript expression analysis of RNA-seq experiments with TopHat and cufflinks. Nat Protoc. (2012) 7:562–78. doi: 10.1038/nprot.2012.016, PMID: 22383036 PMC3334321

[41] Kopylova E Noe L Touzet H. SortMeRNA: fast and accurate filtering of ribosomal RNAs in metatranscriptomic data. Bioinformatics. (2012) 28:3211–7. doi: 10.1093/bioinformatics/bts611, PMID: 23071270

[42] Quast C Pruesse E Yilmaz P Gerken J Schweer T Yarza P. The SILVA ribosomal RNA gene database project: improved data processing and web-based tools. Nucleic Acids Res. (2013) 41:D590–6. doi: 10.1093/nar/gks1219 23193283 PMC3531112

[43] Burge SW Daub J Eberhardt R Tate J Barquist L Nawrocki EP. Rfam 11.0: 10 years of RNA families. Nucleic Acids Res. (2013) 41:D226–32. doi: 10.1093/nar/gks1005 23125362 PMC3531072

[44] Wood DE Salzberg SL. Kraken: ultrafast metagenomic sequence classification using exact alignments. Genome Biol. (2014) 15:R46. doi: 10.1186/gb-2014-15-3-r46 24580807 PMC4053813

[45] Rohart F Gautier B Singh A Le Cao KA. mixOmics: An R package for ‘omics feature selection and multiple data integration. PLoS Comput Biol. (2017) 13:e1005752. doi: 10.1371/journal.pcbi.1005752 29099853 PMC5687754

[46] Lê Cao KA Boitard S Besse P. Sparse PLS discriminant analysis: biologically relevant feature selection and graphical displays for multiclass problems. BMC Bioinformatics. (2011) 12:253. doi: 10.1186/1471-2105-12-253 21693065 PMC3133555

[47] Jiao X Sherman BT Huang da W Stephens R Baseler MW Lane HC. DAVID-WS: a stateful web service to facilitate gene/protein list analysis. Bioinformatics. (2012) 28:1805–6. doi: 10.1093/bioinformatics/bts251, PMID: 22543366 PMC3381967

[48] Love MI Huber W Anders S. Moderated estimation of fold change and dispersion for RNA-seq data with DESeq2. Genome Biol. (2014) 15:550. doi: 10.1186/s13059-014-0550-8 25516281 PMC4302049

[49] Amachawadi RG Purvis TJ Lubbers BV Homm JW Maxwell CL Nagaraja TG. Bacterial flora of liver abscesses in crossbred beef cattle and Holstein steers fed finishing diets with or without tylosin. J Anim Sci. (2017) 95:3425–34. doi: 10.2527/jas.2016.1198, PMID: 28805921

[50] Nagaraja TG Beharka AB Chengappa MM Carroll LH Raun AP Laudert SB. Bacterial flora of liver abscesses in feedlot cattle fed tylosin or no tylosin. J Anim Sci. (1999) 77:973–8. doi: 10.2527/1999.774973x, PMID: 10328365

[51] Amachawadi RG Nagaraja TG. First report of anaerobic isolation of Salmonella enterica from liver abscesses of feedlot cattle. J Clin Microbiol. (2015) 53:3100–1. doi: 10.1128/JCM.01111-15, PMID: 26085617 PMC4540919

[52] Lechtenberg KF Nagaraja TG Leipold HW Chengappa MM. Bacteriologic and histologic studies of hepatic abscesses in cattle. Am J Vet Res. (1988) 49:58–62. PMID: 3354968

[53] Tan ZL Nagaraja TG Chengappa MM. Fusobacterium necrophorum infections: virulence factors, pathogenic mechanism and control measures. Vet Res Commun. (1996) 20:113–40. doi: 10.1007/BF00385634, PMID: 8711893

[54] Pinnell L Whitlow CW Huebner K Bryant T Martin J Belk KE. Not all liver abscesses are created equal: the impact of Tylosin and antibiotic alternatives on bovine liver abscess microbial communities and a first look at Bacteroidetes-dominated communities. Front Microbiol. (2022) 13:882419. doi: 10.3389/fmicb.2022.882419, PMID: 35572696 PMC9094069

[55] Ma YT Li RB Wang JA Jiang WC Yuan XZ Cui JY. ITIH4, as an inflammation biomarker, mainly increases in bacterial bloodstream infection. Cytokine. (2021) 138:155377. doi: 10.1016/j.cyto.2020.155377, PMID: 33348064

[56] Wen N Zhao N Xu H Zhao Y Ma J. Serum inter-alpha-trypsin inhibitor heavy chain 4 in patients with inflammatory bowel disease: correlation with disease risk, inflammation, activity, and its variation after treatment. Ir J Med Sci. (2022) 191:2105–11. doi: 10.1007/s11845-021-02837-3, PMID: 34843071

[57] Pineiro M Alava MA Gonzalez-Ramon N Osada J Lasierra P Larrad L. ITIH4 serum concentration increases during acute-phase processes in human patients and is up-regulated by interleukin-6 in hepatocarcinoma HepG2 cells. Biochem Bioph Res Co. (1999) 263:224–9. doi: 10.1006/bbrc.1999.1349, PMID: 10486281

[58] Conner JG Eckersall PD Wiseman A Aitchison TC Douglas TA. Bovine acute phase response following turpentine injection. Res Vet Sci. (1988) 44:82–8. doi: 10.1016/0034-5288(88)90018-5, PMID: 2453907

[59] Hirvonen J Pyorala S Jousimies-Somer H. Acute phase response in heifers with experimentally induced mastitis. J Dairy Res. (1996) 63:351–60. doi: 10.1017/S0022029900031873, PMID: 8864931

[60] Horadagoda A Eckersall PD Hodgson JC Gibbs HA Moon GM. Immediate responses in serum Tnf-alpha and acute-phase protein concentrations to infection with Pasteurella-Haemolytica A1 in calves. Res Vet Sci. (1994) 57:129–32. doi: 10.1016/0034-5288(94)90094-9, PMID: 7973087

[61] Pineiro M Andres M Iturralde M Carmona S Hirvonen J Pyorala S. ITIH4 (inter-alpha-trypsin inhibitor heavy chain 4) is a new acute-phase protein isolated from cattle during experimental infection. Infect Immun. (2004) 72:3777–82. doi: 10.1128/IAI.72.7.3777-3782.2004, PMID: 15213118 PMC427401

[62] Soeiro C Quilici IR Legoff A Oussalah MB Morin M Alauzet C. Hepatic abscess due to Dialister pneumosintes – a case report. Anaerobe. (2019) 59:35–7. doi: 10.1016/j.anaerobe.2019.05.006, PMID: 31103532

[63] Zhou FQ. Pyruvate in the correction of intracellular acidosis: a metabolic basis as a novel superior buffer. Am J Nephrol. (2005) 25:55–63. doi: 10.1159/000084141, PMID: 15731550

[64] Xu J Wang L. Low T3 syndrome as a predictor of poor prognosis in patients with pyogenic liver abscess. Front Endocrinol. (2019) 10:541. doi: 10.3389/fendo.2019.00541 PMC6691090 31447784

[65] Sheridan P. Thyroid hormones and the liver. Clin Gastroenterol. (1983) 12:797–818. doi: 10.1016/S0300-5089(21)00606-4, PMID: 6616940

[66] Fazio E Bionda A Chiofalo V Crepaldi P Lopreiato V Medica P. Adaptive responses of thyroid hormones, insulin, and glucose during pregnancy and lactation in dairy cows. Animals. (2022) 12:395. doi: 10.3390/ani12111395, PMID: 35681859 PMC9179583

[67] Steinhoff L Jung K Meyerholz MM Heidekorn-Dettmer J Hoedemaker M Schmicke M. Thyroid hormone profiles and TSH evaluation during early pregnancy and the transition period in dairy cows. Theriogenology. (2019) 129:23–8. doi: 10.1016/j.theriogenology.2019.01.023, PMID: 30784791

[68] Lechtenberg KF Nagaraja TG Chengappa MM. Antimicrobial susceptibility of Fusobacterium necrophorum isolated from bovine hepatic abscesses. Am J Vet Res. (1998) 59:44–7. PMID: 9442241

[69] Goussard P Gie RP Steyn F Rossouw GJ Kling S. Pasteurella multocida lung and liver abscess in an immune-competent child. Pediatr Pulmonol. (2006) 41:275–8. doi: 10.1002/ppul.20327, PMID: 16429437

[70] Oh K Inoue T Saito T Nishio C Konishi H. Spinal epidural abscess caused by Pasteurella multocida mimicking aortic dissection: a case report. BMC Infect Dis. (2019) 19:448. doi: 10.1186/s12879-019-4097-x 31113388 PMC6530056

[71] Paasch C Wilczek S Strik MW. Liver abscess and sepsis caused by Clostridium perfringens and Klebsiella oxytoca. Int J Surg Case Rep. (2017) 41:180–3. doi: 10.1016/j.ijscr.2017.10.033, PMID: 29096338 PMC5686218

[72] Law ST Lee MK. A middle-aged lady with a pyogenic liver abscess caused by Clostridium perfringens. World J Hepatol. (2012) 4:252–5. doi: 10.4254/wjh.v4.i8.252, PMID: 22993668 PMC3443708

[73] Miller L. Treat and prevent clostridium in cattle. Progressive Dairy. (2021).

[74] Navarro MA Uzal FA. Pathobiology and diagnosis of clostridial hepatitis in animals. J Vet Diagn Investig. (2020) 32:192–202. doi: 10.1177/1040638719886567, PMID: 31735127 PMC7081508

[75] Wilson BA Ho M. Pasteurella multocida: from zoonosis to cellular microbiology. Clin Microbiol Rev. (2013) 26:631–55. doi: 10.1128/CMR.00024-13, PMID: 23824375 PMC3719492

